# Dance for Adults With Fibromyalgia—What Do We Know About It? Protocol for a Scoping Review

**DOI:** 10.2196/resprot.6873

**Published:** 2017-02-22

**Authors:** Julia Bidonde, Catherine Boden, Angela J Busch, Suelen M Goes, Soo Kim, Emily Knight

**Affiliations:** ^1^ School of Physical Therapy University of Saskatchewan Saskatoon, SK Canada; ^2^ Norwegian Institute of Public Health Oslo Norway; ^3^ Leslie and Irene Dube Health Sciences Library University of Saskatchewan Saskatoon, SK Canada; ^4^ School of Health Sciences University of Western Ontario London, ON Canada

**Keywords:** fibromyalgia, dance, scoping review, physical activity, adults, protocol

## Abstract

**Background:**

Fibromyalgia is a chronic disorder characterized by widespread muscular tenderness, pain, fatigue, and cognitive difficulties. Nonpharmacological treatment options, such as physical activity, are important for people with fibromyalgia. There are strong recommendations to support engagement in physical activity for symptom management among adults with fibromyalgia. Dance is a mode of physical activity that may allow individuals with fibromyalgia to improve their physical function, health, and well-being. Dance has the potential to promote improved pain processing while simultaneously providing the health and social benefits of engaging in physical activity that contributes to symptom management. However, we are unaware of current evidence on dance as a nonpharmacological/physical activity intervention for adults with fibromyalgia.

**Objective:**

The aims of the study are to provide an overview of the extant evidence to understand how dance is used for individuals with fibromyalgia; to examine the extent, range, and nature of research activity in the area; and to determine the value of undertaking a full systematic review.

**Methods:**

Scoping reviews are useful to comprehensively and systematically map the literature and identify key evidence, or research gaps. The search strategy will involve electronic databases including Medline, Embase, Cochrane Library, PsycInfo, Cumulative Index of Nursing and Allied Health Literature (CINAHL), Literature in the Health Sciences in Latin America and the Caribbean (LILACS), Allied and Complementary Medicine (AMED), International Bibliography of Theatre and Dance, Physiotherapy Evidence Database (PEDro), Trip, Proquest Theses/Dissertations, Web of Science, World Health Organization International Clinical Trials Registry Platform, and ClinicalTrials.gov. The study will be mapped in seven stages: (1) identifying the research questions, (2) identifying relevant studies, (3) selecting the studies, (4) charting the data, (5) collating, summarizing and reporting the results, (6) consulting, and (7) disseminating the knowledge.

**Results:**

The search, title, and abstract are now completed; full text screening was carried out and authors are awaiting interlibrary loans and translations. Data extraction will start shortly after full text ‘screening’ is completed. Completion is expected in Fall 2017.

**Conclusions:**

To our knowledge this will be the first attempt to systematically identify knowledge of dance as a potential intervention for adults with fibromyalgia. This scoping review offers a feasible means for describing the evidence specific to dance and fibromyalgia; results will provide unique insights concerning the breadth and depth of literature in the area. An analysis of this body of literature as a whole may reveal new research directions or unknown ways this intervention could strengthen current management approaches of the disease.

## Introduction

### Description of the Condition

Fibromyalgia is a chronic disorder characterized by widespread muscular tenderness, pain, fatigue, and cognitive difficulties [1,2]. Individuals with fibromyalgia may experience sleep disturbances, anxiety, depression [3,4], and difficulty with attention and concentration, as well as a range of gastrointestinal (eg, irritable bowel syndrome) and somatosensory (eg, hyperalgesia, allodynia, paresthesia) symptoms. These symptoms can affect an individual’s quality of life, often negatively impacting family dynamics, capacity and productivity at work, and independence [2]. The diagnosis is often complex requiring a history of typical symptoms over time and the exclusion of somatic diseases by medical examination [1].

Fibromyalgia is common worldwide with the prevalence reported to be 2% to 4% of the general population, with diagnosis in females outnumbering diagnosis in males [1,5]. Insights gained from research in the past several decades implicate numerous factors in its pathophysiology including changes in brain and neural structure and function, muscular physiology, hormonal factors, inflammatory markers, and genetic influences [6]. Mounting evidence shows that individuals with fibromyalgia experience pain differently from the general population because of dysfunctional pain processing in the central nervous system [7].

### Description of the Intervention and How It Might Work

There is vast evidence supporting exercise interventions for individuals with fibromyalgia; in the most recent European League Against Rheumatism guidelines, Macfarlane et al [8] concluded there is a strong recommendation to support both aerobic and resistance training in symptom management for individuals with fibromyalgia. This is in part due to the pain management achieved through physical activity and the low cost and ease of access to physical activity opportunities. Physical activity is defined as any bodily movement produced by skeletal muscles resulting in energy expenditure [9]. Dance, a genre of physical activity, can be a social experience, an artistic expression, or a leisure activity as well as rigorous physical activity. Building on perspectives shared by Beardsley [10], we operationalize dance as a purposeful, deliberate, and expressive motion of the body caused by contraction of the skeletal muscles. Dance may or may not include music; although dance movements could be called “functional” (eg, bending, walking, reaching), the goal of dance movement is the deliberate and purposeful expression of the body itself through movement [11].

There is evidence describing benefits of dance for chronic disease conditions. Dance among individuals with heart failure has demonstrated increased functional and cardiovascular benefits as well as increased motivation for participation [12], quality of life [13], and a reduction in cardiovascular mortality [14] when compared to traditional exercise training. Research shows exercise capacity and quality of life improved with dance in individuals with Parkinson disease [15]. Emotional benefits were seen after dance-based exercise participation in older individuals with or without chronic depression or depressive symptoms [16]. Also, dance enhanced the locomotor ability (ie, movement from one place to another) of individuals with severe rheumatoid arthritis [17]. Other dance genres such as jazz dance [18], Argentine tango [19,20], Turkish folklore [21], Korean traditional dance [22], social dance [23], ballroom dance [24], modern dance [25], waltz [26], and specific designed-exercise dance programs [27] have shown benefits for individuals with a myriad of clinical conditions.

One specific dance-based therapeutic approach common in the literature of individuals with chronic conditions is dance movement therapy (DMT). The American Dance Therapy Association defines DMT as a psychotherapeutic use of movement that furthers the emotional, social, cognitive, and physical integration of the individual. This form of dance has a systematic treatment approach, is goal-oriented, and may include a variety of dance movement methods [28]. Chronic conditions DMT has been used for include cancer [28], schizophrenia [29,30], depression [31], dementia [32,33], and Parkinson disease [15]. We are aware of 3 publications including adults with fibromyalgia [34-36].

Dance contributes to the physical training of balance, coordination, strength, flexibility, aerobic capacity, bone health, and proprioception (ie, knowing where the body is in space). Additionally, dance promotes increased motivation to exercise [37], increased attention and cognitive capacity through increased neural connections and blood flow [38], increased vitality [39], and positive effects on mood [22], everyday competencies, and social life [40]. Dance can also offer auditory, visual and sensory stimulation; motor learning; emotional perception; expression; and interaction. All these features make dance an “enriched environment” which stimulates the brain’s plasticity [40]. Characteristics of dance suggest that it is worth evaluating as a means of relieving fibromyalgia symptoms.

### Pain Processing

Widespread pain and fatigue are hallmark symptoms of fibromyalgia and are known factors limiting an individual’s participation in treatment [41]. During physical activity, the muscular and physiological stress on the body stimulates the release of endorphins, which contributes to the sensation of an activity high and, potentially, a “social high” [42]. Evidence supports that both physical pain (the unpleasant experience that is associated with actual or potential damage to tissue) and social pain (the unpleasant experience that is associated with actual or potential damage to one’s sense of social connection or value) are processed with shared neural circuitry [43]. This supports the hypothesis that experiences in social and physical pain may be similar for the individual, such that individuals experiencing chronic physical pain are more likely to avoid activities for fear of inducing both social and physical pain [43,44]. Therefore, a social activity intervention may lead to improved treatment outcomes for adults with fibromyalgia by improving pain processing.

### Social Bonding and Pain

Dance is an engaging and enjoyable form of physical activity. Group or social dance facilitates social bonds through working in synchrony (performing the same movements at the same time) [42,45]. Synchronization and physical exertion, such as through dance, independently elevate the pain threshold [42]. Moreover, dance can increase self-control, which impacts psychological health and therefore the experience of chronic pain [34]. Dance has the potential to promote improved pain processing while simultaneously providing the health and social benefits of engaging in physical activity that contribute to symptom management for adults with fibromyalgia.

### Why It Is Important to Do This Scoping Review

The authors of this scoping review have worked extensively on synthesizing the evidence of exercise training for adults with fibromyalgia [46-48]. To date, we do not know what evidence exists examining dance for adults with fibromyalgia. As there is a continuous need to offer appropriate nonpharmacological options to people with fibromyalgia, and after contemplating various systematic approaches available for reviewing the literature, we chose to undertake a scoping review as the best method to understand the evidence around dance for adults with fibromyalgia. We wish to examine the extent, range, and nature of research activity in the area and determine the value of undertaking a full systematic review.

## Methods

### Overview

Scoping review methodology is particularly useful for examining the breadth of the research in a specific topic area. Also, scoping reviews are useful to comprehensively and systematically map the literature and identify key evidence or research gaps. Unlike most synthesis reviews, scoping reviews do not narrow the review to specific research designs. Nonetheless, this type of review is rigorous and methodical in its approach to examining the extent, range, and nature of research activity in a particular field [49-52] while encompassing both empirical and conceptual research with openly framed questions.

In designing the protocol for this scoping review, we drew primarily upon Arksey and O’Malley’s seminal work [49] on a 6-stage scoping review framework. Adaptations (including the addition of a seventh step) were driven by an intention to develop a feasible approach for reviewing the body of literature.

### Stage 1. Identifying the Research Questions

Following Arksey and O’Malley’s suggestion, we followed an iterative process for developing the research questions. We continued doing this as we became increasingly familiar with the literature. We realized the need for an iterative process and first ran a trial search.

Our intention to comprehensively examine and map the evidence on dance in adults (ie, 18 years or older) with fibromyalgia prompted us to develop the following initial questions:

What is known from the literature about dance for adults with fibromyalgia (eg, definition of dance, participant characteristics)?

What type of dance is commonly used (eg, traditional mainstream, adapted, dance to music) and what are the characteristics of dances reported (frequency, time, length, etc)? Who is in charge of the instruction, and what is the setting in which dance occurs?

What type of publications are reporting dance, what is the quality of the publications, and what are the main outcomes measured and reported?

Do studies report the acceptability, feasibility, and applicability of dance for clinical practice?

Have studies reported any challenges or limitations upon implementation of a dance class/intervention?

### Stage 2: Identifying Relevant Studies

The aim of this scoping review will be to comprehensively address ‘the above’ broad research questions; however, parameters are required to guide the search strategy.

#### Eligibility Criteria

The following inclusion criteria will be used to guide the search and review the articles:

- Published in any language (for publications that are not in a language mastered by the review team [English, Norwegian, Swedish, Icelandic, Spanish, German, Portuguese, French] individuals proficient in the language or translation software will be used)
- Human subjects
- Adults aged 18 years and older with fibromyalgia
- Publications that target adults with fibromyalgia of any gender or ethnicity in any setting (private practice, clubs, community association) and type of dance
- Publications including research projects, pilot experience, and protocols
- Scope limited to include published literature (ie, peer-reviewed journals, books or book chapters, dissertations, guidelines) and grey literature
- Concepts of dance/therapy/movement and fibromyalgia evident either in title or abstract during screening phase

Explicit exclusion criteria identified:

- Publications with a population that is not exclusively adults with fibromyalgia or we cannot isolate the results for adults with fibromyalgia
- Publications in which individuals are nonactive participants (ie, they are observers only)

The nature of a scoping review is to include multiple forms of evidence and not exclusively randomized controlled trials. Our inclusion criteria were established to identify and include research reports of participation in dance and exclude research reports of observation of dance. Therefore, self-report data, such as published case studies or reports in the grey literature, of participants engaging in dance as related to fibromyalgia symptoms would meet inclusion criteria.

#### Databases

An experienced information specialist will establish and test the search strategy. Based on her expertise and the outline of this project, she will select keywords and controlled vocabulary terms to maximize sensitivity and specificity within the search. She will be instrumental in choosing and applying search terms to comply with databases in the health and social sciences. The complete and final search strategy will be provided in a follow-up publication. Upon completion, the results from each database will be documented, and the references will be imported into a bibliographic management software to eliminate duplicates. References will be imported to a review software for screening.

Databases used:

- Medline in-process and other nonindexed citations (Ovid)—1946 to present
- Embase and Embase Classic (Ovid)—1947 to present
- Cochrane Library (Wiley)
- PsycINFO (Ovid)—1806 to present
- Cumulative Index of Nursing and Allied Health Literature (CINAHL) (EBSCO)—1937 to present
- Literature in the Health Sciences in Latin America and the Caribbean (Literatura Latino Americana em Ciências da Saúde, LILACS)
- Allied and Complementary Medicine (AMED) (Ovid)—1985 to present
- International Bibliography of Theatre and Dance (EBSCO)—1984 to present
- Physiotherapy Evidence Database (PEDro)
- Trip
- ProQuest Theses and Dissertations—1997 to present
- Web of Science Core Collection (Thomson Reuters)—1900 to present
- World Health Organization International Trial Registry Portal
- ClinicalTrials.gov

Searching other resources:

- We will search the bibliographies of relevant studies and reviews.
- Corresponding authors of previously found dance randomized controlled trials will be contacted regarding their knowledge of ongoing studies or groups involved in the area.
- An a priori set of fibromyalgia associations will be selected and their associations’ webpages will be screened for annual reports or findings that these associations produce based on their own research, which will be retrieved.

### Stage 3. Selecting the Studies

We will use a 2-stage selection process. In the first instance, 2 reviewers will independently screen citations and abstracts for inclusion. At this stage, uncertainties from the reviewers will not automatically eliminate the record. We will determine final inclusion at the second level (full text screening). A third reviewer will arbitrate in cases where there is disagreement at the final stage. All authors will be trained in software use, and a predetermined and piloted criteria will be followed at both stages.

The full text of all papers identified as having potential for inclusion will be requested. Non- English articles will be translated. Data will be extracted from papers included by independent review authors. A Preferred Reporting Items for Systematic Reviews and Meta-Analyses (PRISMA) flow diagram [53] will be used to report final numbers upon completion of the scoping review.

### Stage 4. Charting the Data

We will collect and sort information from the selected full text articles (see Table 1). We will use standardized data extraction forms created and piloted by the team for this purpose. Team members will train in data extraction to standardize the process and ensure consistency of the data extraction process. We will examine the charting consistency with the questions and purpose. Additional categories may emerge during the data collection process, in which case, in consultation with the team, we will adapt and restructure the forms.

**Table 1:** Data to be extracted

Data	Details to be extracted (if available)
Publication summary	Author, year, title, publication type, inclusion/exclusion, country, language of publication
Population	Total sample size, age, gender, diagnosis, years since diagnosis, baseline characteristics, comorbidities, medication, diagnostic criteria
Intervention	Objective/type (ie, leisure, training, complementary) Descriptor such as: frequency, intensity, length, mode, setting, instructor qualifications, follow-up, use of music, dance alone, in-group, with partner
Setting	Community, hospital, club, university, etc. Cost and equipment
Outcomes	Any outcomes: symptoms (eg, pain, fatigue, sleep), psychosocial (eg, depression, self-awareness, mood, self-esteem), physical function (eg, physical health, range of motion, cardiovascular, strength, flexibility), health-related quality of life, relationships, and communication (isolation, verbalization, family support), withdrawals. Adverse events, harms, or related terms: challenges, limitations, barriers, injuries, exacerbations
Other	Effectiveness Adherence to intervention such as acceptability, feasibility, applicability for clinical practice

Given the authors’ backgrounds in systematic review of interventions for adults with fibromyalgia, the appraisal of included studies will be restricted (if found) to those scientific publications explicitly addressing intervention effectiveness. This may provide direction for future research.

### Stage 5. Collating, Summarizing, and Reporting the Results

The unique purpose of a scoping review is to aggregate the findings and present an overview.

We plan to do the following:

- Map results (main sources, quantity, and quality of evidence available) from the literature.
- Provide a descriptive summary: extracted data from all included publications will be summarized to describe the use of dance in adults with fibromyalgia. Because this is a scoping review, there is no principal summary measure. However, if possible, the following analyses will be completed:
  - Descriptive statistics will be used to summarize the data. Frequencies and percentages will be used to describe nominal data.
  - Conceptual definitions will be subject to a comparative analysis where verbatim ‘extracts’ will be coded by review authors. The purpose will be to identify the dimensions and properties of each definition as well as their relationships with other components. This analysis will involve identification of dimensions of the concept of dance, recurrent themes, variations, contradictions, and connections.
  - If possible, we will use computer-assisted clustering techniques to present the information in graphical form (eg, bubble plot, word cloud) [54,55].
- A glossary of terms will be created to clarify definitions found in the literature.

We will follow and adapt PRISMA reporting guidelines for systematic reviews [53], PRISMA equity [56], and a PRISMA harms checklist [57] to accurately report the results and analysis summary.

### Step 6: Consulting

With the aim to ensure applicability and usability of results, the findings of this scoping review will be shared throughout the protocol writing, collating, and summarizing phases with the consumers associated with the team. They will provide their comments and thoughts throughout the scoping review’s duration. We will also engage research experts in dance, fibromyalgia, and synthesis methods to review the project protocol and provide objective feedback in findings and final reporting. The fibromyalgia and physical activity team (led by AJB) will be asked to validate our findings and provide feedback and guidance on the completion of the final manuscript. All responses and opinions will be integrated into the study.

### Step 7: Disseminating the Knowledge

Although not part of Arksey’s framework, we believe it is important to make the content of this scoping review available to clinicians and consumers with the goal of increasing awareness of the literature and helping to make evidence-informed choices for clinical management of fibromyalgia. Some of the steps we plan to take include writing a scientific publication, presenting the results at a conference, distributing a plain language summary report to self-help groups and organizations working with individuals with fibromyalgia, posting a summary of the results to the fibromyalgia and exercise team website, and exploring the dance4healing app as a venue to distribute our results. Additionally, we plan to develop teaching material from this scoping review (eg, case study) to be used in chronic disease management courses in health and rehabilitation programs involving undergraduate and graduate students.

## Results

The search, title, and abstract are now completed; full text screening was carried out and authors are awaiting interlibrary loans and translations. Data extraction will start shortly after full text ‘screening’ is completed Completion is expected in Fall 2017.

## Discussion

A large body of evidence from the past decades supports the use of physical activity as one of the main nonpharmacological interventions for adults with fibromyalgia. Exercise training (eg, aquatic, resistance, aerobic) is often part of the overall management of fibromyalgia, decreasing peoples’s symptoms and improving their quality of life. However, our understanding of dance use in this population is limited. To our knowledge this will be the first attempt to systematically identify knowledge of dance as a potential intervention for adults with fibromyalgia.

This scoping review offers a feasible means for synthesizing the evidence specific to dance and fibromyalgia; results will provide unique insights concerning the breadth and depth of literature in the area. We anticipate we will be able to identify research trends and potential gaps specific to our research questions as well as novel ideas for primary research concerning this intervention. An analysis of this body of literature as a whole may reveal new research directions or unknown ways this intervention could strengthen current management approaches of the disease.

## Abbreviations

AMED: Allied and Complementary Medicine
CINAHL: Cumulative Index of Nursing and Allied Health Literature
DMT: dance movement therapy
LILACS: Literature in the Health Sciences in Latin America and the Caribbean
PEDro: Physiotherapy Evidence Database
PRISMA: Preferred Reporting Items for Systematic Reviews and Meta-Analyses
